# Global prevalence of fluoroquinolone resistance in common *Campylobacter* species associated with human infections

**DOI:** 10.1016/j.nmni.2025.101687

**Published:** 2025-12-26

**Authors:** Zeinab Fagheei Aghmiyuni, Mohammad Hossein Ahmadi, Kasra Javadi

**Affiliations:** a Department of Microbiology, Faculty of Medicine, Shahed University, Tehran, Iran; b Infectious Diseases and Tropical Medicine Research Center, Health Research Institute, Babol University of Medical Sciences, Babol, Iran; c Department of Laboratory Sciences, School of Allied Medical Sciences, Babol University of Medical Sciences, Babol, Iran

**Keywords:** *Campylobacter* spp., Fluoroquinolone resistance, Prevalence, Nalidixic acid, Norfloxacin, Ciprofloxacin

## Abstract

**Background:**

Fluoroquinolones have long been the primary choice in treating campylobacteriosis, yet rising resistance threatens their effectiveness. Understanding global resistance rates in common *Campylobacter* species is vital for guiding treatment, improving antimicrobial stewardship, and develop effective interventions for controlling this public health concern. This study aimed to investigate the global prevalence of fluoroquinolone resistance in common clinical *Campylobacter* species.

**Methods:**

We searched PubMed/MEDLINE, Web of Science, Scopus, and Embase for relevant studies published from 1992 to 2025 using the keywords: *Campylobacter* spp., *Campylobacter*, fluoroquinolone, quinolone, resistance, susceptible, and sensitive. A total of from 630 articles were found. After removing duplicates, irrelevant studies, non-English/review/non-human studies, and those with insufficient data/species-level resistance reporting, 81 eligible studies retained for final analysis. The data were then subjected to a comprehensive meta-analysis.

**Findings:**

The mean resistance rates (95 % CI) to fluoroquinolones for *C. jejuni* were 53 % (norfloxacin), 51.4 % (ciprofloxacin), and 51.2 % (nalidixic acid), and for *C. coli* were 27.1 %, 54.7 %, and 56.9 %, respectively. The included studies mainly sampled stool and rectal swabs. Agar dilution and disk diffusion were common susceptibility testing methods. High heterogeneity (I^2^ >50 %) was common among the studies. Begg's test showed no publication bias (P > 0.05), but Egger's test detected bias in some ciprofloxacin/nalidixic acid resistance analyses (P < 0.05).

**Conclusions:**

Given the relatively high fluoroquinolone resistance observed, restricting fluoroquinolone use in animal agriculture to preserve treatment options as well as seeking for new therapeutic alternatives and enhancing global surveillance are essential for effectively managing the infections caused by *Campylobacter* species, especially in high-risk populations.

## 1 Introduction

*Campylobacter*, a genus of Gram-negative bacteria, is widely present in the digestive tracts of several animal species and is the primary bacterial source of diarrhea and foodborne diseases [1]. These bacteria, which have a spiral form and may move using either a single flagellum or two flagella, frequently have a curved or comma-shaped rod-like appearance [2].

There are more than 39 species under the *Campylobacter* genus [3]. *Campylobacter jejuni* and *Campylobacter coli* are the primary etiological agents of human disease, responsible for around 90 % and 5 % of cases, respectively [4,5]. Additional species, including *C*. *lari*, *C*. *upsaliensis*, *C*. *hyointestinalis*, *C. concisus*, and *C*. *sputorum*, are becoming recognized as possible disease-causing agents. *C*. *fetus*, which causes spontaneous abortions in cattle and sheep, may also cause opportunistic infections in humans. They may also be found in domesticated animals like cats and dogs [3,6].

*Campylobacter* is well recognized as the predominant bacterial agent responsible for human gastroenteritis on a global scale [6]. It is widespread in livestock species such as poultry, cattle, pigs, sheep, and ostriches [7]. A comprehensive study determined the combined prevalence of *Campylobacter* species from various sources in Ethiopia to be 10.2 % [8,9]. The reported prevalence of gastroenteritis caused by *Campylobacter* in Singapore was 7.6 cases per 100,000 individuals [10]. Most people infected with *Campylobacter* may recover without antibiotics. However, it may be life-threatening for infants, the elderly, and those with weakened immune systems [11]. Thus, antibiotics may be required for those experiencing severe sickness or those who are susceptible to acquiring severe illness [12].

Fluoroquinolones and macrolides are often recommended as the first-line therapy for diarrhea caused by *Campylobacter* infections [13]. Nevertheless, a noteworthy surge in ciprofloxacin resistance has been seen in the strains of both *C*. *coli* and *C*. *jejuni* [14]. When *Campylobacter* species develop resistance to fluoroquinolones, other therapies such as azithromycin and erythromycin, which belong to the macrolide class of antibiotics, are often used [15]. However, the increasing prevalence of macrolide resistance is a significant medical issue. Although macrolide resistance in *C*. *jejuni* remains relatively low, it has grown more frequently in *C*. *coli* [16].

It is important to highlight that antibiotic resistance, particularly to fluoroquinolones, poses a significant challenge to the effective treatment of *Campylobacter* infections [17]. In 2017, *Campylobacter* was classified as a high-priority pathogen on the World Health Organization's (WHO) list of bacteria requiring urgent development of new antibiotics due to the increasing resistance to fluoroquinolones in the strains, worldwide [18]. Quinolone/fluoroquinolone resistance primarily arises from chromosomal mutations affecting efflux pumps, DNA gyrase, and/or topoisomerase IV [19,20]. Transferable mechanisms called plasmid-mediated quinolone resistance (PMQR) have emerged in recent decades, especially in Gram-negative bacteria [20,21]. Fluoroquinolone-resistant Enterobacteriaceae frequently harbor PMQR determinants such as qnr (*qnrA, qnrB, qnrC, qnrD, qnrS, and qnrVC*), *qep, oqxAB*, or *aac* (6′)-*Ib-cr* genes [20,22]. The aminoglycoside acetyltransferase AAC (6′)-Ib enzyme, which confers resistance to tobramycin, kanamycin, and amikacin, was first identified in *Klebsiella pneumoniae* isolates in 1986, and several variants have since been described [20,23,24]. This enzyme reduces ciprofloxacin susceptibility by N-acetylation of its piperazinyl amine [20,25]. AAC (6′)-Ib-cr is a bifunctional acetyltransferase that modifies both aminoglycosides and quinolones. This enzyme is widely reported worldwide in Gram-negative bacteria, particularly *Enterobacteriaceae* from human, farm animal, and environmental sources, with both plasmid and chromosomal encoding, indicating a high potential for dissemination [20,26].

This study, systematically examines global fluoroquinolone resistance in *Campylobacter* species linked to human infections from 1992 to 2025. Its main goal is to compile current data and analyze worldwide resistance trends, addressing gaps in previous researches. By focusing on resistance mechanisms, geographical distribution, and factors driving resistance emergence and spread, it offers a fresh perspective. Additionally, the study enhances understanding of therapeutic challenges and potential solutions by comparing its findings with similar epidemiological and clinical reviews.

## 2 Material and methods

### 2.1 Study selection criteria

This meta-analysis examined antibiotic resistance in *Campylobacter* species (including *C. jejuni*, *C. coli*, and *C. fetus*) to norfloxacin, ciprofloxacin, and nalidixic acid. Included studies met these criteria: (1) provided quantitative species-level resistance data, (2) involved human samples (feces, rectal swabs, blood, or other clinical specimens), (3) were published between 1992 and 2025, and (4) had full texts available in English. Excluded were animal or environmental studies, review articles, conference abstracts, and studies reporting resistance only at the genus level.

### 2.2 Search strategy and study selection

The databases including PubMed/MEDLINE, Web of Science, Scopus, and Embase were searched for studies published between 1992 and 2025 using the keywords “*Campylobacter* spp. AND *Campylobacter* AND fluoroquinolone AND quinolone AND resistance AND Susceptible OR sensitive”. The references of retrieved articles were also reviewed manually.

### 2.3 Data extraction

Data extraction was conducted independently by one researcher using a standardized form. Extracted data included: (1) study details (author, publication year, country), (2) sample demographics, (3) clinical sample types, (4) laboratory methods for antibiotic resistance detection, and (5) quantitative resistance data for each antibiotic. When the proportion of susceptible isolates was reported, the number of resistant *Campylobacter* isolates was calculated by subtracting the susceptibility percentage from 100, dividing by 100, and multiplying by the total isolates. Conversely, when the resistant proportion was given, the number of susceptible isolates was found by dividing that proportion by 100 and multiplying by the total isolates.

### 2.4 Assessment of study quality and publication bias

Calculation of study quality and publication bias was conducted using the Newcastle-Ottawa Scale for cohort studies and the PRISMA checklist for meta-analyses reporting [27]. Publication bias was evaluated through Begg's and Egger's tests, alongside funnel plot analysis.

### 2.5 Statistical analysis

Statistical analyses were conducted using a Comprehensive Meta-Analysis software (version 3.7z, Bio stat). Pooled prevalence with 95 % confidence intervals (CIs) was calculated using a random-effects model. Heterogeneity was assessed using the I^2^ statistic and Q test, with I^2^ >50 % and Q test p < 0.05 indicating significant heterogeneity. Subgroup analyses by geographical region, laboratory methods, and clinical sample type were accomplished when significant heterogeneity was detected. Sensitivity analysis was conducted by excluding individual studies to assess result stability.

Bivariate linear regression, was conducted using GraphPad Prism version 9.5 to analyze the temporal trends in *C. jejuni* and *C. coli* resistance to ciprofloxacin and nalidixic acid from 1992 to 2025, by reporting average resistance rates with 95 % CIs. For antibiotics with sufficient data, the regression equation and R^2^ value were also calculated.

## 3 Results

A total of 630 articles were collected for assessment. In the first screening, 191 articles were excluded based on title evaluation. Of these, 37 were duplicate publications of the same study, and 154 had titles irrelevant to our research. During the second assessment, 300 papers were eliminated because they were either in a language other than English, classified as review articles, or derived from non-human samples. After thoroughly evaluating the full texts, 58 studies were ruled out due to insufficient data, being reported only at the genus level, or lacking information on antibiotic resistance at the species level. As a result, 81 articles published between 1992 and 2025 were selected for the final analysis (Fig. 1 and Table 1).

**Fig. 1:**
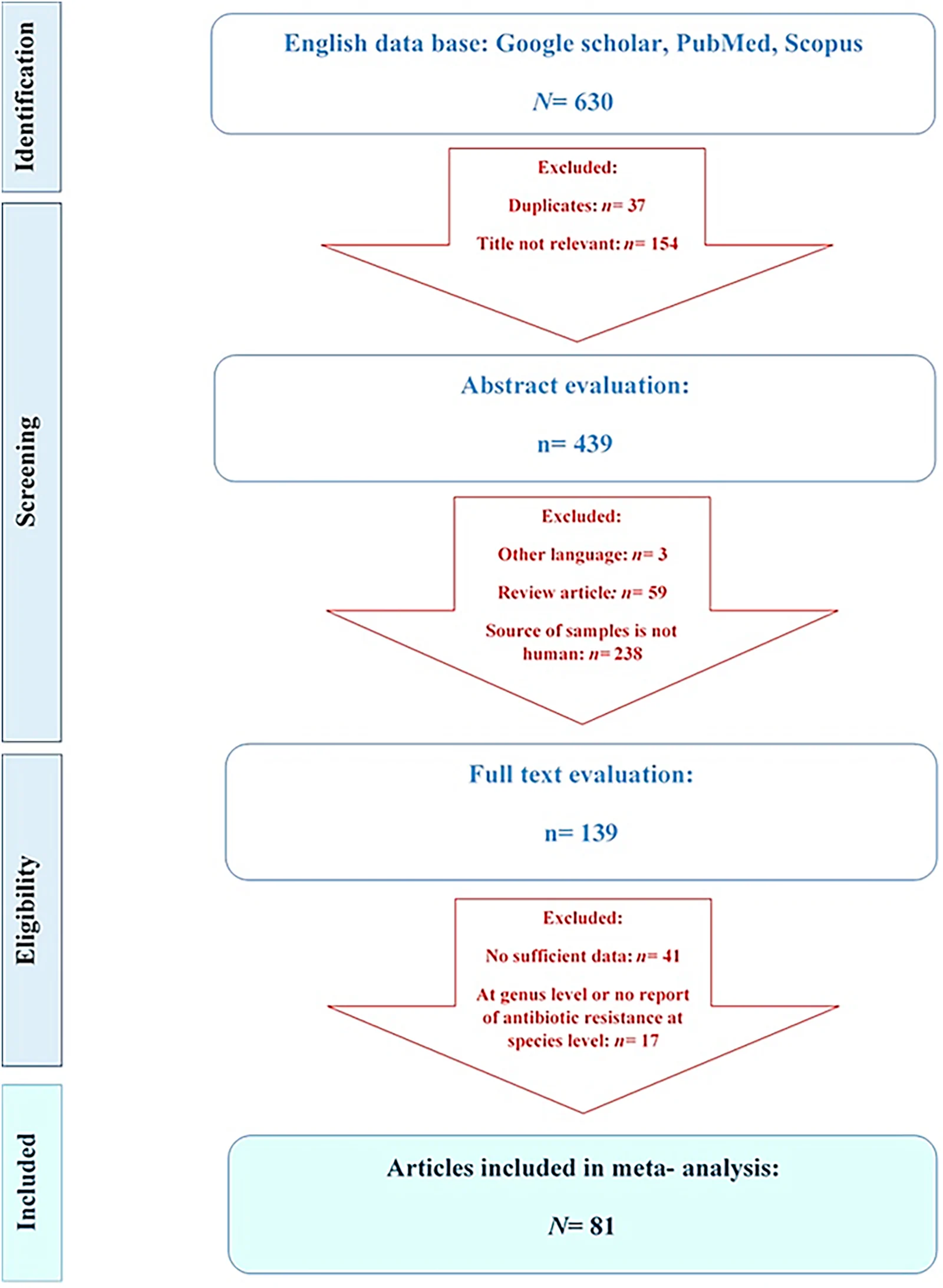
Flow chart of the literature search, systematic review and study selection.

**Table 1:** Studies included in meta-analysis after final evaluation.

Author (Reference)	Pub. Year	Country	Source/specimen	Species	No. of tested isolates	No. of resistant isolate (%)			Method of susceptibility testing	Disease/complication/participants
						NOR	CIP	NA		
Chiurtu et al. [23]	2025	Romania	Stool	C. j C. c	46 17	NM	43 (93.4) 15 (88.2)	NM	Disc diffusion	Childhood *Campylobacter* Infections Before and After the COVID-19 Pandemic
Matos et al. [24]	2025	Northern Portugal	Stool	C. j C. c	157 140	NM	151 (96.2) 134 (95.7)	NM	Disc diffusion	Patients diagnosed with *Campylobacter* sp.
Emara et al. [25]	2025	Egypt	Stool	C. j C. c	10 5	NM	4 (40) 2 (40)	NM	Disc diffusion	Gastroenteritis patients
Bos et al. [26]	2024	France	Stool/blood	C. j C. c C. f	165 25 7	NM	101 (61.2) 17 (68) 3 (42.8)	NM	Disc diffusion	Patients with campylobacteriosis in Kidney Transplant Recipients
Betts et al. [27]	2024	Gambia	Stool	C. j	45	NM	2 (4.4)	2 (4.4)	Disc diffusion	Children under five with moderate-to-severe diarrhea and healthy Controls
Wei et al. [28]	2024	Taiwan	Stool	C. j C. c	221 36	NM	209 (94.4) 29 (81)	209 (94.4) 29 (81)	commercial Sensititre Campylobacter CAMPY AST panel plates (Thermo Fisher Scientific Co.)	Diarrheal patients
Wu et al. (a) [29]	2024	China	Stool	C. j C. c	34 6	NM	28 (82.3) 5 (83.3)	31 (91.18) 5 (83.3)	Agar dilution method	Diarrheal patients
Cooper et al. [30]	2024	Peru	Stool	C. j C. c	105 72	NM	72 (68.5) 51 (70.8)	NM	Disc diffusion	Patients with diarrhea and asymptomatic
Baltoiu et al. [31]	2024	Romania	Stool	C. j	66	NM	47 (71.2)	NM	Disc diffusion	Patients with acute diarrheal disease
Pablo et al. [32]	2024	Spain	Stool	C. j	114	NM	103 (90.3)	NM	Disc diffusion	Patients with diarrhea
Alobaidy et al. [33]	2024	Turkey	Rectal swab	C. j C. c	42 9	NM	40 (95.23) 7 (77.7)	NM	Disc diffusion	Patients with gastroenteritis
Schiaffino et al. [34]	2024	Peru	Stool	C. j C. c	97 67	NM	68 (70.10) 52 (77.6)	NM	Disc diffusion	Pediatrics with and without diarrhea
García-Fernandez et al. [35]	2024	Italy	Stool and blood	C. j C. c	102 31	NM	31 (30.39) 3 (9.68)	NM	Broth microdilution and agar dilution	Based on a systematic voluntary network
Kim et al. [36]	2024	South Korea	Stool and rectal swabs	C. j C. c	361 10	NM	298 (82.6) 10 (100)	231 (64.1) 9 (90)	Broth microdilution	Diarrheal patients
Baek et al. [37]	2023	South Korea	Blood	C. j C. c C. f	38 11 15	NM	26 (68) 7 (63.6) 1 (6.7)	NM	Disc diffusion	Patients with *Campylobacter* bacteraemia
Wu et al. (b) [38]	2023	China	Stool	C. j C. c	9 2	NM	9 (100) 1 (50)	9 (100) 1 (50)	Agar dilution	Diarrheal patients
e Zeller-Péronnet et al. [39]	2023	Germany	Stool	C. j C. c	44 24	NM	36 (81.8) 19 (79.2)	NM	Broth microdilution (Sensititre)	Gastroenteritis patients
Gao et al. [40]	2023	China	Stool	C. j C. c	761 130	NM	734 (96.45) 130 (100)	720 (94.61) 128 (98.46)	Broth microdilution	Outpatients with diarrhea
Ford et al. [41]	2023	United States	Stool	C. j C. c	13138 1500	NM	3422 (26.05) 532 (35.5)	NM	Broth microdilution (Sensititre)	NS/Patients
Ghielmetti et al. [42]	2023	Switzerland	Stool	C. j	340	NM	169 (49.8)	169 (49.8)	Broth microdilution (Sensititre)	Patients with gastroenteritis
Zhang et al. [43]	2023	China	Stool	C. j	184	NM	141 (76.6)	140 (76.1)	Agar dilution	NS/Patients
Aydin et al. [44]	2023	Turkey	Rectal swab	C. j	77	NM	33 (42.85)	35 (45.45)	E-test	Acute gastroenteritis.
Sasaki et al. [45]	2023	Japan	Stool	C. j	151	NM	70 (46.4)	71 (47)	Broth microdilution	Enteritis patients
Quino et al. [46]	2022	Peru	Stool and rectal swabs	C. j C. c	108 21	NM	102 (94.4) 21 (100)	102 (94.4) 21 (100)	Agar microdilution	NS/Patients
Metreveli et al. [47]	2022	Georgia	Stool	C. j C. c	47 10	NM	41 (87) 9 (90)	NM	Broth microdilution	Children with diarrhea
Elhadidy et al. [48]	2020	Belgium	Stool	C. j	199	NM	111 (55.8)	112 (56.3)	Broth microdilution (Sensititre)	Diarrheal patients
Zhang et al. [49]	2020	China	Stool	C. j C. c	200 36	NM	189 (94.50) 34 (94.44)	184 (92) 32 (88.89)	Agar dilution	Patients with acute diarrhea
Elhadidy et al. [50]	2019	Belgium	Stool	C. c	14	NM	12 (85.7)	12 (85.7)	Broth microdilution (Sensititre)	Patients with gastroenteritis symptoms
Meistere et al. [51]	2019	Latvia	Stool	C. j	18	NM	13 (72.2)	13 (72.2)	Broth microdilution	Routinely tested human stool samples from sporadic cases of gastroenteritis were analyzed for gastrointestinal bacterial pathogens
Aksomaitiene et al. [52]	2019	Lithuania	Stool	C. j	101	NM	89 (88.1)	NM	Agar dilution	Human clinical cases
Yamada et al. [53]	2019	Japan	Stool	C. j	430	NM	165 (38.3)	NM	Broth microdilution	NS/Patients
Kayman et al. [54]	2019	Turkey	Stool	C. j	152	NM	113 (74.3 %)	NM	E-test	Acute gastroenteritis/Patients
Schiaffino et al. [55]	2019	Peru	Stool	C. j	588	NM	455 (77.4)	383 (64.9)	Disc diffusion	Diarrheal and surveillance fecal samples/children enrolled in the MAL-ED cohort of Peru
Devi et al. [56]	2019	Australia	Stool	C. j	117	NM	16 (13.7)	NM	Disc diffusion	Patients with diarrhea
Marotta et al. [57]	2019	Italy	Stool	C. j	51	NM	39 (76.4)	38 (74.5)	Broth microdilution	Gastroenteritis/Acute campylobacteriosis
García-Fernández et al. [58]	2018	Italy	Stool, blood, urine, unidentified source	C. j C. c C. f	176 41 1	NM	134 (76) 29 (70) 0	NM	Disc diffusion	Gastroenteritis, Campylobacteriosis/pediatric and adult patients
Wieczorek et al. [59]	2018	Poland	Rectal swabs	C. j	155	NM	132 (85.2)	131 (84.5)	Broth microdilution (Sensititre)	Diarrhea
Sierra-Arguello et al. [60]	2018	Brazil	Rectal swabs	C. j C. c	11 10	1 (9.1) 4 (40)	1 (9.1) 4 (40)	1 (9.1) 4 (40)	Broth microdilution	Patients with gastroenteritis
Li et al. [61]	2018	China	Stool	C. j C. c	24 2	NM	21 (87.5) 2 (100)	22 (91.7) 2 (100)	Agar dilution	Campylobacteriosis/Abdominal pain, Nausea and Vomiting, Dehydration/acute diarrhea among outpatients
Szczepanska et al. [62]	2017	Poland	Stool	C. j C. c	92 7	NM	60 (65.2) 5 (71.4)	NM	E-test	Children aged 0–4 with diarrhea
Cha et al. [63]	2016	United States	Stool	C. j	94	NM	18 (19.15)	18 (19.15)	Broth microdilution (Sensititre)	Patients with Campylobacteriosis
Zhou et al. [64]	2016	China	Stool	C. j	203	NM	176 (86.6)	176 (86.6)	Agar dilution	Patients with diarrhea
Olkkola et al. [65]	2016	Finland	Stool	C. j	95	NM	8 (8.4)	8 (8.4)	Broth microdilution	NS/Patient
Kim et al. [66]	2015	Korea	Stool	C. j	63	NM	61 (96.8)	63 (100)	Broth microdilution (Sensititre)	Patients with diarrhea
Abay et al. [67]	2014	Turkey	Stool	C. j	100	NM	NM	84 (84)	Disc diffusion	Patients with diarrhea
Igimi et al. [68]	2014	Japan	Stool	C. j	422	129 (31)	122 (29)	120 (28)	Disc diffusion	Diarrhea/patients
Mukherjee et al. [69]	2013	India	Stool	C. j	142	138 (97)	138 (97)	138 (97)	Disc diffusion	Hospitalized Patients with Diarrhea
Pollett et al. [70]	2012	Peru	Stool	C. j C. c	3856 554	NM	3084 (79.9) 291 (52.5)	NM	Disc diffusion	Ns/patients
Liao et al. [71]	2012	Taiwan	Blood	C. j C. c C. f	3 15 6	NM	3 (100) 10 (66.7) 3 (50)	NM	Agar dilution	Patients with intra-abdominal infection (54.2 %), followed by primary bacteremia (41.7 %), and cellulitis (4.2 %). Abdominal pain, nausea, and diarrhea were the most common symptoms.
Sonnevend et al. (a) [72]	2011	Hungary	Stool	C. j	147	NM	100 (68)	101 (68.7)	Agar dilution	Campylobacteriosis/patients
Said et al. [73]	2010	Egypt	Stool or rectal swabs	C. j C. c	100 18	NM	89 (89) 14 (78)	89 (89) 14 (78)	E-test	Diarrhea
Kittl et al. [74]	2010	Switzerland	Stool	C. j	136	NM	51 (37.5)	51 (37.5)	Broth microdilution (Sensititre)	Gastroenteritis/Patients
Feodoroff et al. [75]	2009	Finland	Stool	C. j C. c	166 26	NM	84 (51) 13 (50)	NM	Broth microdilution	Diarrhea, fever, vomiting, bloody stools
Rożynek et al. [76]	2008	Poland.	Stool	C. j C. c	89 14	NM	53 (59.6) 8 (57.1)	53 (59.6) 8 (57.1)	E-test	Children with diarrhea
Bakeli et al. [77]	2008	Japan	Stool	C. j	523	NM	106 (20.2)	120 (22.9)	Agar dilution	Diarrheal diseases
Feizabadi et al. [78]	2007	Iran	Stool	C. j C. c	29 5	NM	17 (58.6) 4 (80)	22 (75.8) 5 (100)	Disc diffusion	Campylobacteriosis/Diarrheic children
Levesque et al. [79]	2007	Canada	Stool	C. j	254	NM	23 (9)	NM	Agar dilution	NS/Patients
Wardak et al. [80]	2007	Poland	Stool	C. j C. c	102 6	NM	57 (55.8) 4 (66.6)	57 (55.8) 4 (66.6)	E-test	Patients with diarrhea/Bloody diarrhea, fever, vomiting, and abdominal pain
Unicomb et al. [81]	2006	Australia	Stool	C. j	574	NM	14 (2.4)	35 (6.1)	Agar dilution	Patients with diarrhea
Sonnevend et al. (b) [82]	2006	United Arab Emirates	Stool	C. j	41	NM	35 (85.4)	35(85.4)	Agar dilution	Diarrhea or Gastroenteritis
Guévremont et al. [83]	2006	Canada	Stool	C. j C. c	23 16	NM	2 (9) 3 (19)	NM	Agar dilution	Campylobacteriosis/24 originated from clinical cases of sporadic diarrhea
Cardinale et al. [84]	2006	Senegal	Stool	C. j	10	NM	3 (30)	3 (30)	E-test	Epidemiologically unrelated domestic patients
Norström et al. [85]	2006	Norway abroad	Stool	C. j	567	NM	270 (47.6)	278 (49)	E-test	Campylobacteriosis
Lehtopolku et al. [86]	2005	Finland	Stool	C. j	226	134 (59.3)	134 (59.3)	NM	Agar dilution	Patients with gastroenteritis
Boonmar et al. [87]	2005	Thailand	Stool and blood	C. j	50	NM	41 (82)	39 (78)	Disc diffusion	NS/Patients
Tracz et al. [88]	2005	Canada	Stool	C. j	104	NM	3 (3)	4 (4)	Disc diffusion	Enteritis/diarrhea, bloody diarrhea, pain, fever, vomiting
Gibreel et al. [89]	2004	Canada	Stool	C. j	203	NM	4 (2)	5 (2.5)	Agar dilution	NS/Patients
Sharma et al. [90]	2003	Australia	Stool	C. j	180	NM	5 (2.9)	6 (3.4)	Agar dilution	Patients with diarrhea
Hakanen et al. [91]	2003	Finland	Stool	C. j	376	NM	174 (46)	NM	Agar dilution	NS/Patients
Luber et al. [92]	2003	Germany	Stool	C. j C. c	133 31	NM	34 (25.5) 7 (22.5)	NM	Broth microdilution (Sensititre)	Patients with diarrhea
Gaudreau and Gilbert [93]	2003	Canada	Stool	C. j	245	NM	65 (26.5)	NM	Agar dilution	NS/Patients
Bodhidatta et al. [94]	2003	Thailand	Stool samples or rectal swabs	C. j C. c	138 36	NM	122 (88) 35 (97)	NM	Agar dilution	Bloody stool, abdominal colic/Children with acute dysentery.
Sáenz et al. [95]	2000	Spain	Stool	C. j C. c	537 58	NM	403 (75) 41 (70.7)	NM	Agar disk diffusion	NS/Patients
Aquino et al. [96]	2002	Brazil	Stool	C. j C. c	11 4	2 (18.2) 1 (25)	2 (18.2) 1 (25)	NM	Disc diffusion	Children with diarrhea
Thwaites and Frost [97]	1999	North West England and Wales	Stool	C. j C. c C. l	5401 376 25	NM	567 (10.5) 82 (21.8) 25 (100)	799 (14.8) 101 (26.9) 25 (100)	Agar dilution	Gastrointestinal infections/Patients
Li et al. [98]	1998	Taiwan	Stool	C. j C. c	73 20	NM	38 (52) 15 (75)	58 (79) 17 (85)	E-Test	Enterocolitis/pediatrics patients
Ruiz et al. [99]	1998	Spain	Stool	C. j	313	NM	219 (69.9)	219 (69.9)	Agar dilution	NS/Patients
Gallardo et al. [100]	1998	Spain	Stool	C. j	24	NM	3 (12.5)	3 (12.5)	Disc diffusion	Traveler's Diarrhea/Patients with diarrhea
Gaudreau et al. [101]	1998	Canada	Stool	C. j	291	NM	23 (7.9)	26 (8.9)	Agar dilution	NS/Patients
Sanchez et al. [102]	1994	Spain	Stool	C. j C. c	230 42	80 (34.9) 6 (14.3)	73 (31.8) 4 (9.5)	91 (39.6) 10 (23.8)	Agar dilution	NS/Patients
Reina et al. [103]	1992	Spain	Stool	C. j C.c	614 58	NM	66 (10.7) 7 (12.06)	73 (11.8) 8 (13.8)	Agar dilution	Diarrhea/pediatric patients (under 12 years old)

NM: not measured, NS: not specified, C. j: *Campylobacter jejuni*, C. c: *Campylobacter coli*, C. f: *Campylobacter fetus, NA*: nalidixic acid, CIP: Ciprofloxacin, NOR: Norfloxacin.

The included studies were conducted across 30 countries, with 33 in Europe, 26 in Asia, 8 in North America, 7 in South America, and 4 in Africa. Among the 81 reviewed articles, one focused solely on *C*. *jejuni*, 42 on *C*. *coli*, 34 on both *C*. *jejuni* and *C*. *coli*, and four on *C*. *jejuni*, *C*. *coli*, and *C*. *fetus*, simultaneously. Sample sources included stool (67 studies), rectal swabs (4 studies), blood (2 studies), and a combination of stool, blood, urine, or unspecified specimens (8 studies).

The included studies evaluated antimicrobial susceptibility to norfloxacin, ciprofloxacin, and nalidixic acid for both *C*. *jejuni* (6, 79, and 48 studies, respectively) and *C*. *coli* (3, 40, and 18 studies, respectively). Additionally, ciprofloxacin susceptibility was tested for *C*. *fetus* in 3 studies. The most commonly used method for *Campylobacter* susceptibility testing was agar dilution (25 studies), followed by disk diffusion (24 studies) and broth microdilution (20 studies). E-test was applied in 9 studies, while one study used both broth microdilution and agar dilution, and another employed *Campylobacter* CAMPY AST panel plates.

The range (minimum-maximum) of antibiotic resistance rates and the pooled resistance rates for *C*. *jejuni*, *C*. *coli*, and *C*. *fetus* to norfloxacin, ciprofloxacin, and nalidixic acid are presented in Table 2. The average resistance rates for *C*. *jejuni* were 53 % for norfloxacin, 51.4 % for ciprofloxacin, and 51.2 % for nalidixic acid. In case of *C*. *coli*, the average resistance rates were 27.1 %, 54.7 %, and 56.9 %, respectively. The average resistance rate for *C*. *fetus* to ciprofloxacin was 28.3 %.

**Table 2:** Meta-analysis results for resistance rate of *Campylobacter* isolates in included studies.

Clinical isolate	Antibiotic	Number of studies	Resistance rate (%) (95 % CI)			Heterogeneity test		Begg's test^d^*p*-value (two-tailed)		Egger's test^e^*p*-value (two-tailed)
			Min	Max	Pooled^c^ (range)	*I*^2^ (%)	*p*-value	a	b	
***C. jejuni***	Norfloxacin Ciprofloxacin Nalidixic acid	6 79 48	9.1 2.9 2.5	97 100 100	0.4 (0.2–0.6) 0.5 (0.4–0.6) 0.5 (0.4–0.6)	95.7 99.1 98.7	<0.001 <0.001 <0.001	0.5 0.2 0.2	0.7 0.2 0.2	0.6 0.002 0.002
***C. coli***	Norfloxacin Ciprofloxacin Nalidixic acid	3 40 18	14.3 9.5 13.8	40 100 100	0.2 (0.1–0.3) 0.6 (0.5–0.7) 0.7 (0.5–0.8)	37.3 91.6 90.6	0.2 <0.001 <0.001	0.6 0.5 0.7	1.0 0.6 0.8	0.6 0.001 0.0007
***C. fetus***	Norfloxacin Ciprofloxacin Nalidixic acid	0 3 0	– 6.7 –	– 50 –	– 0.3 (0.09–0.6) –	– 55.9 –	– 0.1 –	– 0.6 –	– 1.0 –	– 0.2 –

a Kendall's tau without continuity correction.

b Kendall's tau with continuity correction.

c Pooled resistance rate (based on random effects, except for norfloxacin resistance in *C. coli* (fixed effect)).

d Begg and Mazumdar rank correlation.

e Egger's regression intercept.

A high level of heterogeneity was observed across all studies (I^2^>50 %, P < 0.05), except for those assessing norfloxacin resistance in *C*. *coli* isolates, which showed lower heterogeneity (I^2^ = 37.3 %, P = 0.2). Begg's tests did not indicate publication bias (P > 0.05). However, Egger's tests indicated evidence of publication bias in studies on ciprofloxacin resistance in *C*. *jejuni* isolates (P = 0.002), nalidixic acid resistance in *C*. *jejuni* isolates (P = 0.002), ciprofloxacin resistance in *C*. *coli* isolates (P = 0.001), and nalidixic acid resistance in *C*. *coli* isolates (P = 0.0007) (Table 2).

Time trend analysis using Pearson correlation showed a significant increase in *C. jejuni* and *C. coli* resistance to ciprofloxacin and nalidixic acid from 1992 to 2025 (*C. jejuni* vs. Ciprofloxacin, r = 0.679, p = 0.0001; *C. jejuni* vs. Nalidixic acid, r = 0.702, p = 0.0002; *C. coli* vs. Ciprofloxacin, r = 0.704, p = 0.0004; *C. coli* vs. Nalidixic acid, r = 0.753, p = 0.0029), indicating a strong correlation with publication year. Fig. 2 shows time trend resistance rates for ciprofloxacin and nalidixic acid from 1992 to 2025.

**Fig. 2:**
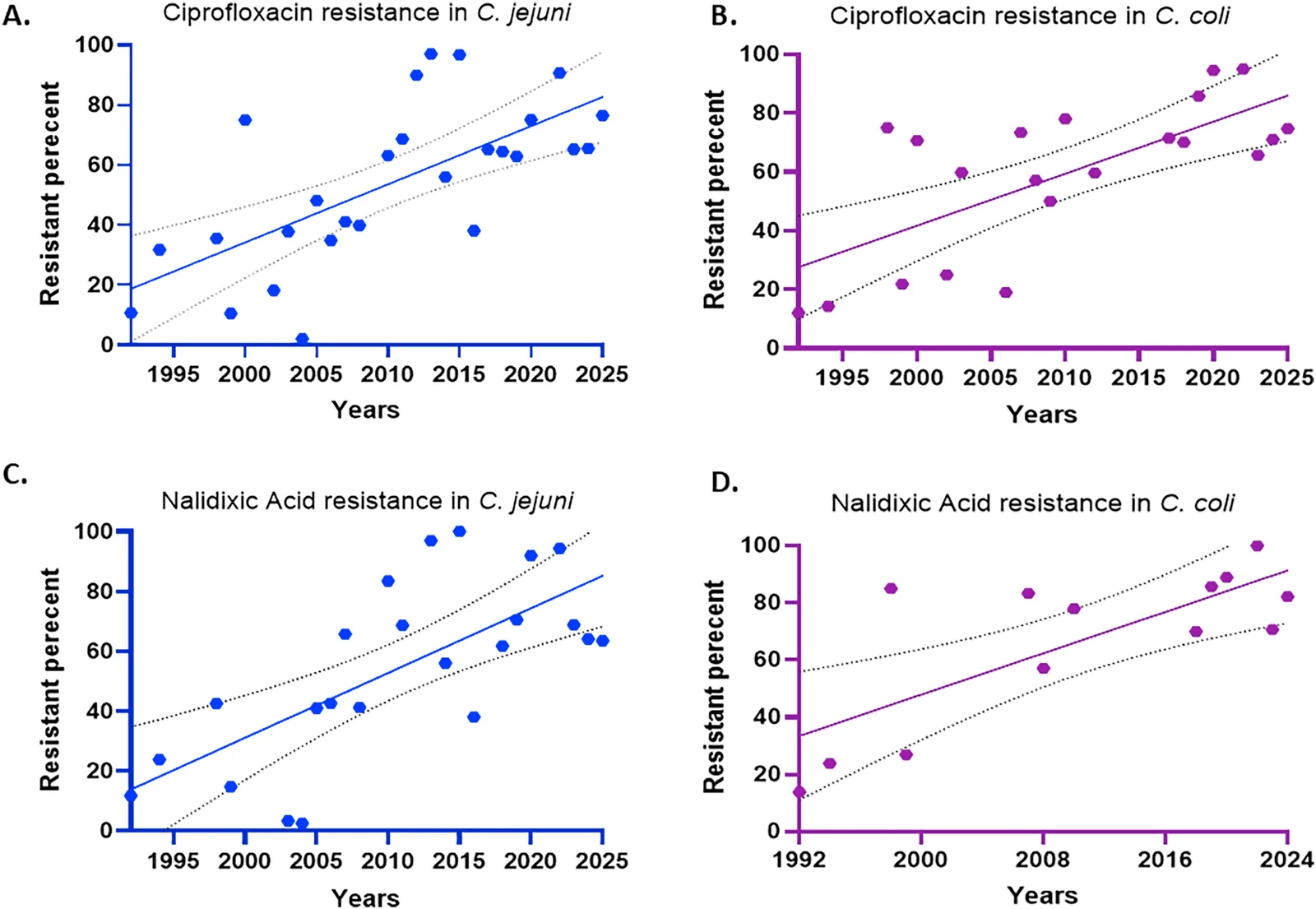
Resistance rates to ciprofloxacin in *C. jejuni* and (A) and *C. coli* (B), and nalidixic acid in *C. jejuni* (C), and *C. coli* (D), from 1992 to 2025. Dashed lines indicate 95 % confidence intervals (CI) (A: 0.4031–0.8418, B: 0.3922–0.8713, C: 0.4092–0.8646, D: 0.3466–0.9218).

## 4 Discussion

The present study confirmed that fluoroquinolone resistance in *Campylobacter* spp. has risen to high levels globally over the past 30 years, with pooled resistance exceeding 50 % in *C. jejuni* and *C. coli*.

### 4.1 Global resistance patterns and species-specific variations

Our meta-analysis revealed a high combined resistance in both *C. jejuni* and *C. coli* to ciprofloxacin and nalidixic acid. Time trend analysis revealed a clear increase in fluoroquinolone resistance from 1992 to 2025. Strong positive correlations between resistance rates and publication year for both *C. jejuni* and *C. coli* indicate sustained selection pressure driving resistance development. The increasing antibiotic resistance in *Campylobacter* spp., due to development of resistance mechanisms, diminishes the treatment efficacy and brings about significant health concerns. Humans mainly acquire infections by consuming contaminated animal-based foods, particularly undercooked poultry and unpasteurized milk and dairy products [104,105]. Fluoroquinolones, along with macrolides and tetracyclines, are among the three most commonly used antimicrobials for treating systemic *Campylobacter* infections; gentamicin (an aminoglycoside) is also occasionally used [106,107].

In this study, *C. coli* exhibited greater resistance to nalidixic acid compared to *C. jejuni*, which supports existing research indicating that *C. coli* may develop resistance more easily due to genomic characteristics or selective pressures in livestock [108,109]. Quinolone/fluoroquinolone resistance, primarily in *C. coli,* is established through chromosomal mutations in the *gyrA* gene and further evolves via acquisition of *aac(6′)-Ib-cr* on a genetic background already abundant in the wild-type *aac(6′)-Ib* gene [20]. Antibiotic resistance in *Campylobacter*, especially to fluoroquinolones and macrolides, poses a significant public health risk. A study of *Campylobacter* isolates from cattle in USA revealed high fluoroquinolone resistance in *C. jejuni* (35.4 %) and in *C. coli* (74.4 %), exceeding previous reports. All fluoroquinolone-resistant *C. coli* had a Thr-86-Ile GyrA mutation, while *C. jejuni* isolates had other *GyrA* mutations in addition to Thr-86-Ile. Notably, fluoroquinolone-resistant *C. coli* isolates displayed similar PFGE (pulsed field gel electrophoresis) patterns and the ST-1068 sequence type, regardless of origin or isolation time, suggesting clonal expansion drives their spread. In contrast, *C. jejuni* isolates were genetically diverse [109].

In the present study, *C. fetus* exhibited lower ciprofloxacin resistance (28.3 %), possibly due to less fluoroquinolone exposure in its systemic infection niche compared to enteric colonization. *C*. *fetus* is a pathogen mainly linked to fertility issues in sheep and cattle, and it can cause serious infections in humans that need antimicrobial treatment [110]. Understanding antimicrobial resistance in *C. fetus* is crucial, as it causes bacteremia with a 14 % fatality rate and may require extended antimicrobial treatment [110,111]. *C. fetus* is inherently resistant to nalidixic acid [110,112].

### 4.2 Geographical and methodological variation

Our analysis of the included studies revealed geographically variable antibiotic resistance patterns. While uneven data distribution across regions, with most studies originating from Europe and Asia and limited data from Africa and South America, hampered comprehensive comparisons, the observed differences may reflect variations in research capacity, surveillance systems, and dissemination practices rather than true epidemiological differences [113]. Regional variations could stem from differing antibiotic use policies in livestock and poultry, regulatory frameworks, and clinical prescribing practices.

Variations in antibiotic susceptibility testing methods (agar dilution, disk diffusion, broth microdilution, etc.) significantly affected our findings and their interpretation. This methodological diversity introduces variability in resistance classification due to differing sensitivities and specificities. The high statistical heterogeneity (I^2^ >50 %) likely reflects both true biological variation and methodological differences between the studies [114]. The low level of heterogeneity for *C. coli* regarding norfloxacin resistance (I^2^ = 37.3 %) is related to the limited studies included. Publication bias, detected by Egger's test, may suggest an overrepresentation of certain resistance patterns due to selective publication of significant or concerning findings [115].

### 4.3 Public health implications and urgent priorities

The increasing resistance to fluoroquinolones threatens their use as first-line treatment for suspected *Campylobacter* infections [116]. The increasing antimicrobial resistance in *Campylobacter* spp. is concerning [117], particularly given the potential for poultry-to-human transmission, necessitating judicious antibiotic use and *Campylobacter* reduction in the poultry industry [116]. Furthermore, antibiotic tolerance in *C*. *jejuni* is significant, as exposure to ciprofloxacin or tetracycline, common in livestock farming, induces tolerance and promotes fluoroquinolone resistance [118].

The results of this study underscore the urgent need for comprehensive strategies to combat *Campylobacter* resistance. These should include enhanced surveillance to track resistance trends, stricter regulation of antibiotics in animal feed, antimicrobial stewardship in human medicine, and the development of rapid diagnostic tests to identify resistant strains and optimize antibiotic use [119].

### 4.4 Limitations and directions for future research

Our meta-analysis had some limitations that may affect the results. It did not include studies on the prevalence of resistance genes or the molecular mechanisms behind resistance differences among *Campylobacter* species. Additionally, there were a few studies on other *Campylobacter* species causing human infections and on resistance to norfloxacin. By focusing solely on published studies, there is a risk of publication bias; indeed, our Egger's test indicated that such bias may exist, suggesting that studies reporting lower resistance rates are underrepresented in the literature. Language bias may also be present, as we excluded non-English studies, which may have omitted data from countries where relevant research is published in local languages. Finally, the high heterogeneity observed across studies, likely due to actual geographic and methodological variation, means that pooled prevalence values should be interpreted as approximate indicators of resistance levels rather than precise estimates. Nonetheless, the consistently high resistance rates across diverse settings strongly indicate a substantial and widespread global issue. To address this, future research should involve prospective, multicenter studies using standardized methods to explore resistance differences and assess intervention effectiveness in reducing resistance rate.

## 5 Conclusions

The present study reveals a global rise in fluoroquinolone resistance in *C. jejuni* and *C. coli*, with the latter showing the highest levels. Resistance is projected to grow from 1992 to 2025 due to antibiotic overuse in humans and livestock. Geographic variations in resistance patterns indicate inconsistent monitoring and regulation. The findings underscore the urgent need to restrict fluoroquinolone use in animal agriculture, standardize susceptibility testing, and enhance global surveillance. Without these measures, treatment options for severe campylobacteriosis, particularly in vulnerable groups, will continue to diminish.

## CRediT authorship contribution statement

**Zeinab Fagheei Aghmiyuni:** Writing – original draft, Methodology, Investigation, Data curation. **Mohammad Hossein Ahmadi:** Writing – review & editing, Supervision, Methodology, Formal analysis, Conceptualization. **Kasra Javadi:** Writing – original draft, Methodology, Investigation.

## Ethics approval and consent to participate

Not applicable.

## Data availability statement

The original contributions presented in the study are included in the article/supplementary material; further inquiries can be directed to the corresponding authors.

## Declaration of competing interest

The authors declare that they have no known competing financial interests or personal relationships that could have appeared to influence the work reported in this paper.

## References

[1] Same R.G., Tamma P.D. (2018). Campylobacter infections in children. Pediatr Rev.

[2] Fono-Tamo U.E.K., Chukwu M.O., Ubomba-Jaswa E., Obi C., Dewar J.B., Abia A.L.K. (2020). Emerging and reemerging bacterial pathogens of humans in environmental and hospital settings. Curr Microbiol Res Afr.

[3] Huang H., Garcia M.M. (2022;2:1-30). Isolation and identification of campylobacter spp. from food and food-related environment. Campylobacter.

[4] Ben Romdhane R., Merle R. (2021). The data behind risk analysis of Campylobacter jejuni and Campylobacter coli infections. Curr Top Microbiol Immunol.

[5] Murugesan M., Abraham D., Samuel P., Ajjampur S.S. (2022). Campylobacter diarrhea in children in south Asia: a systematic review. Indian J Med Microbiol.

[6] Igwaran A., Okoh A.I. (2019). Human campylobacteriosis: a public health concern of global importance. Heliyon.

[7] Shange N., Gouws P., Hoffman L.C. (2019). Campylobacter and arcobacter species in food-producing animals: prevalence at primary production and during slaughter. World J Microbiol Biotechnol.

[8] Zenebe T., Zegeye N., Eguale T. (2020). Prevalence of campylobacter species in human, animal and food of animal origin and their antimicrobial susceptibility in Ethiopia: a systematic review and meta-analysis. Ann Clin Microbiol Antimicrob.

[9] Ammar A.M., El-Naenaeey E.-S.Y., Abd El-Hamid M.I., El-Gedawy A.A., Elmalt R.M. (2021). Campylobacter as a major foodborne pathogen: a review of its characteristics, pathogenesis, antimicrobial resistance and control. J Microbiol Biotechnol Food Sci.

[10] Liu F., Lee S.A., Xue J., Riordan S.M., Zhang L. (2022). Global epidemiology of campylobacteriosis and the impact of COVID-19. Front Cell Infect Microbiol.

[11] Zhang S., Shi J., Li X., Tiwari A., Gao S., Zhou X., Sun X., Luby S., O'Brien J.W., Coin L. (2023). Wastewater-based epidemiology of campylobacter spp.: a systematic review and meta-analysis of influent, effluent, and removal of wastewater treatment plants. Sci Total Environ.

[12] Maćkiw E., Korsak D., Rzewuska K., Tomczuk K., Rożynek E. (2012). Antibiotic resistance in Campylobacter jejuni and Campylobacter coli isolated from food in Poland. Food Control.

[13] Dai L., Sahin O., Grover M., Zhang Q. (2020). New and alternative strategies for the prevention, control, and treatment of antibiotic-resistant campylobacter. Transl Res.

[14] Sproston E.L., Wimalarathna H.M., Sheppard S.K. (2018). Trends in fluoroquinolone resistance in campylobacter. Microb Genom.

[15] Tang M., Zhou Q., Zhang X., Zhou S., Zhang J., Tang X., Lu J., Gao Y. (2020). Antibiotic resistance profiles and molecular mechanisms of campylobacter from chicken and pig in China. Front Microbiol.

[16] Liao Y.-S., Chen B.-H., Teng R.-H., Wang Y.-W., Chang J.-H., Liang S.-Y., Tsao C.-S., Hong Y.-P., Sung H.-Y., Chiou C.-S. (2022). Antimicrobial resistance in Campylobacter coli and Campylobacter jejuni from human campylobacteriosis in Taiwan, 2016 to 2019. Antimicrob Agents Chemother.

[17] Inglis G.D., Taboada E.N., Boras V.F. (2021). Rates of fluoroquinolone resistance in domestically acquired Campylobacter jejuni are increasing in people living within a model study location in Canada. Can J Microbiol.

[18] Portes A.B., Panzenhagen P., Pereira dos Santos A.M., Junior C.A.C. (2023). Antibiotic resistance in campylobacter: a systematic review of South American isolates. Antibiotics.

[19] Authority EFS, Prevention ECfD, Control (2021). The european union one health 2019 zoonoses report. EFSA J.

[20] Gharbi M., Abbas M.A.S., Hamrouni S., Maaroufi A. (2023). First report of aac(6′)-Ib and aac(6′)-Ib-cr variant genes associated with mutations in gyrA encoded fluoroquinolone resistance in avian Campylobacter coli strains collected in Tunisia. Int J Mol Sci.

[21] Hooper D.C., Jacoby G.A. (2015). Mechanisms of drug resistance: quinolone resistance. Ann NY Acad Sci.

[22] Yang H.Y., Nam Y.S., Lee H.J. (2014). Prevalence of plasmid‐mediated quinolone resistance genes among ciprofloxacin‐nonsusceptible Escherichia coli and Klebsiella pneumoniae isolated from blood cultures in Korea. Can J Infect Dis Med Microbiol.

[23] Vakulenko S.B., Mobashery S. (2003). Versatility of aminoglycosides and prospects for their future. Clin Microbiol Rev.

[24] Tolmasky M., Roberts M., Woloj M., Crosa J. (1986). Molecular cloning of amikacin resistance determinants from a Klebsiella pneumoniae plasmid. Antimicrob Agents Chemother.

[25] Hidalgo-Grass C., Strahilevitz J. (2010). High-resolution melt curve analysis for identification of single nucleotide mutations in the quinolone resistance gene aac (6′)-Ib-cr. Antimicrob Agents Chemother.

[26] Martinez-Martinez L., Eliecer Cano M., Manuel Rodríguez-Martínez J., Calvo J., Pascual A. (2008). Plasmid-mediated quinolone resistance. Expert Rev Anti Infect Ther.

[27] Moher D., Liberati A., Tetzlaff J., Altman D.G. (2009). Preferred reporting items for systematic reviews and meta-analyses: the PRISMA statement. BMJ.

[28] Chiurtu C., Mocanu E., Nitu B.F., Iancu A.M., Mihai C.M., Cambrea M.A., Mihai R., Mavrodin M., Pînzaru A.D., Stoicescu R.M. (2025). The impact of antibiotic resistance in childhood campylobacter infections before and after the COVID-19 pandemic in the southeast region of Romania. Antibiotics.

[29] Matos M.I., Rocha R., Pinto J., Guimarães A., Lino R., Rocha M.H., Patacho M., Duro R., Santos L., Almeida J. (2025). Zoonotic foodborne infections in a tertiary healthcare setting: clinical and epidemiological aspects of campylobacter and salmonella infection in northern Portugal in 2010–2020. Microbiol Res.

[30] Emara M.S., Ammar A.M., Abdelwahab A.M.O., Elgdawy A.A., Abdelkhalek A., Pet E., Dumitrescu G., Ahmadi M., Abd El-Aziz N.K. (2025). Green tea extract (Theaceae; Camellia sinensis L.): a promising antimicrobial, anti-quorum sensing and antibiofilm candidate against multidrug-resistant campylobacter species. Antibiotics.

[31] Bos F., Gueneau R., Crepin T., Tinévez C., Taton B., Couzi L., Moreau K., Schvartz B., Perrin P., Gatault P., Scemla A., Chatelet-Pouliquen V., Levi C., Kamar N., Lanternier F., Lanotte P., Neau D., Merville P., Lehours P., Puges M., Kaminski H. (2024). Epidemiology of campylobacter species infection in kidney transplant recipients: a retrospective multicentric case-control study in France. Open Forum Infect Dis.

[32] Betts M., Dione M., Ikumapayi U.N., Senghore M., Lamin M., Foster-Nyarko E., Jaffali J., Panchalingam S., Nataro J.P., Kotloff K.L., Levine M.M., Saha D., Hossain J., Kwambana-Adams B., Antonio M. (2024). Genetic diversity and antimicrobial resistance of Campylobacter jejuni isolates from Gambian children under five with moderate-to-severe diarrhoea and healthy controls. bioRxiv.

[33] Wei H.-L., Liao Y.-S., Chen B.-H., Teng R.-H., Wang Y.-W., Chang J.-H., Chiou C.-S. (2024). Antimicrobial resistance and genetic relatedness among Campylobacter coli and Campylobacter jejuni from humans and retail chicken meat in Taiwan. J Glob Antimicrob Resist.

[34] Wu X., Liping C., Dong F., Yan W., Shen Y., Ji L. (2024). Molecular typing and antimicrobial susceptibility profiles of Campylobacter jejuni and Campylobacter coli isolates from patients and raw meat in huzhou, China, 2021–2022. PLoS One.

[35] Cooper Kerry K., Mourkas E., Schiaffino F., Parker Craig T., Pinedo Vasquez Tackeshy N., Garcia Bardales Paul F., Peñataro Yori P., Paredes Olortegui M., Manzanares Villanueva K., Romaina Cachique L., Silva Delgado H., Hitchings Matthew D., Huynh S., Sheppard Samuel K., Pascoe B., Kosek Margaret N. (2024). Sharing of cmeRABC alleles between C. coli and C. jejuni associated with extensive drug resistance in campylobacter isolates from infants and poultry in the Peruvian amazon. mBio.

[36] Baltoiu M., Gradisteanu Pircalabioru G., Cristea D., Sorokin M., Dragomirescu C.C., Stoica I. (2024). Genetic diversity, virulence, and antibiotic resistance determinants of Campylobacter jejuni isolates in Romania. Pathogens.

[37] Fernández-Palacios P., Galán-Sánchez F., Casimiro-Soriguer Carlos S., Jurado-Tarifa E., Arroyo F., Lara M., Chaves J.A., Dopazo J., Rodríguez-Iglesias Manuel A. (2024). Genotypic characterization and antimicrobial susceptibility of human Campylobacter jejuni isolates in southern Spain. Microbiol Spectr.

[38] Alobaidy H.A.K., Ayvaz A., Sağiroğlu P., Atalay M.A. (2024). Diagnosis of campylobacter spp. isolates and their antimicrobial susceptibility patterns. Indian J Microbiol.

[39] Schiaffino F., Parker C.T., Paredes Olortegui M., Pascoe B., Manzanares Villanueva K., Garcia Bardales P.F., Mourkas E., Huynh S., Peñataro Yori P., Romaina Cachique L., Gray H.K., Salvatierra G., Silva Delgado H., Sheppard S.K., Cooper K.K., Kosek M.N. (2024). Genomic resistant determinants of multidrug-resistant campylobacter spp. isolates in Peru. J Glob Antimicrob Resist.

[40] Garcia-Fernandez A., Janowicz A., Marotta F., Napoleoni M., Arena S., Primavilla S., Pitti M., Romantini R., Tomei F., Garofolo G. (2024). Antibiotic resistance, plasmids, and virulence-associated markers in human strains of Campylobacter jejuni and Campylobacter coli isolated in Italy. Front Microbiol.

[41] Kim S.Y., An D., Jeong H., Kim J. (2024). Antimicrobial susceptibility patterns and genetic diversity of campylobacter spp. isolates from patients with diarrhea in South Korea. Microorganisms.

[42] Baek Y.J., Song J.E., Kim E.J., Choi H., Sohn Y., Jeon Y.D., Lee E.H., Ahn J.Y., Jeong S.J., Ku N.S. (2024). Trends, clinical characteristics, antimicrobial susceptibility patterns, and outcomes of campylobacter bacteraemia: a multicentre retrospective study. Infection.

[43] Wu X., Ji L., Shen Y., Chen L., Xu D., Dong F. (2023). Genomic characterization of campylobacter isolates in huzhou, China. PLoS One.

[44] Zeller-Péronnet V., Bretschneider N., Lausch J., Hanifi N., Pavlovic M., Zarske M., Luu H.Q., Busch U., Stingl K., Huber I. (2023). Multiplex real-time PCR for the detection of tetracycline, ciprofloxacin, and erythromycin resistance determinants from human and foodborne Campylobacter jejuni and Campylobacter coli. Microorganisms.

[45] Gao F., Tu L., Chen M., Chen H., Zhang X., Zhuang Y., Luo J., Chen M. (2023). Erythromycin resistance of clinical Campylobacter jejuni and Campylobacter coli in shanghai, China. Front Microbiol.

[46] Ford L., Healy J.M., Cui Z., Ahart L., Medalla F., Ray L.C., Reynolds J., Laughlin M.E., Vugia D.J., Hanna S. (2023). Open forum infect dis.

[47] Ghielmetti G., Seth-Smith H.M., Roloff T., Cernela N., Biggel M., Stephan R., Egli A. (2023). Whole-genome-based characterization of Campylobacter jejuni from human patients with gastroenteritis collected over an 18 year period reveals increasing prevalence of antimicrobial resistance. Microb Genom.

[48] Zhang D., Zhang X., Lyu B., Tian Y., Huang Y., Lin C., Yan H., Jia L., Qu M., Wang Q. (2023). Genomic analysis and antimicrobial resistance of Campylobacter jejuni isolated from diarrheal patients—beijing municipality, China, 2019–2021. China CDC Weekly.

[49] Aydin F., Kayman T., Abay S., Hizlisoy H., Saticioğlu İ.B., Karakaya E., Sahin O. (2023). MLST genotypes and quinolone resistance profiles of Campylobacter jejuni isolates from various sources in Turkey. Int J Food Microbiol.

[50] Sasaki Y., Ikeda T., Yonemitsu K., Kuroda M., Ogawa M., Sakata R., Uema M., Momose Y., Ohya K., Watanabe M. (2023). Antimicrobial resistance profiles of Campylobacter jejuni and salmonella spp. isolated from enteritis patients in Japan. J Vet Med Sci.

[51] Quino W., Caro-Castro J., Hurtado V., Flores-León D., Gonzalez-Escalona N., Gavilan R.G. (2022). Genomic analysis and antimicrobial resistance of Campylobacter jejuni and Campylobacter coli in Peru. Front Microbiol.

[52] Metreveli M., Bulia S., Tevzadze L., Tsanava S., Zarske M., Goenaga J.C., Preuß S., Lomidze G., Koulouris S., Imnadze P. (2022). Comparison of antimicrobial susceptibility profiles of thermotolerant campylobacter spp. isolated from human and poultry samples in Georgia (Caucasus). Antibiotics.

[53] Elhadidy M., Ali M.M., El-Shibiny A., Miller W.G., Elkhatib W.F., Botteldoorn N., Dierick K. (2020). Antimicrobial resistance patterns and molecular resistance markers of Campylobacter jejuni isolates from human diarrheal cases. PLoS One.

[54] Zhang P., Zhang X., Liu Y., Jiang J., Shen Z., Chen Q., Ma X. (2020). Multilocus sequence types and antimicrobial resistance of Campylobacter jejuni and C. coli isolates of human patients from beijing, China, 2017–2018. Front Microbiol.

[55] Elhadidy M., Miller W.G., Arguello H., Álvarez‐Ordóñez A., Dierick K., Botteldoorn N. (2019). Molecular epidemiology and antimicrobial resistance mechanisms of Campylobacter coli from diarrhoeal patients and broiler carcasses in Belgium. Transbound Emerg Dis.

[56] Meistere I., Ķibilds J., Eglīte L., Alksne L., Avsejenko J., Cibrovska A., Makarova S., Streikiša M., Grantiņa-Ieviņa L., Bērziņš A. (2019). Campylobacter species prevalence, characterisation of antimicrobial resistance and analysis of whole-genome sequence of isolates from livestock and humans, Latvia, 2008 to 2016. Euro Surveill.

[57] Aksomaitiene J., Ramonaite S., Tamuleviciene E., Novoslavskij A., Alter T., Malakauskas M. (2019). Overlap of antibiotic resistant Campylobacter jejuni MLST genotypes isolated from humans, broiler products, dairy cattle and wild birds in Lithuania. Front Microbiol.

[58] Yamada K., Saito R., Muto S., Sasaki M., Murakami H., Aoki K., Ishii Y., Tateda K. (2019). Long-term observation of antimicrobial susceptibility and molecular characterisation of Campylobacter jejuni isolated in a Japanese general hospital 2000–2017. J Glob Antimicrob Resist.

[59] Kayman T., Abay S., Aydin F., Şahin O. (2019). Antibiotic resistance of Campylobacter jejuni isolates recovered from humans with diarrhoea in Turkey. J Med Microbiol.

[60] Schiaffino F., Colston J.M., Paredes-Olortegui M., François R., Pisanic N., Burga R., Peñataro-Yori P., Kosek M.N. (2019). Antibiotic resistance of campylobacter species in a pediatric cohort study. Antimicrob Agents Chemother.

[61] Devi A., Mahony T.J., Wilkinson J.M., Vanniasinkam T. (2019). Antimicrobial susceptibility of clinical isolates of Campylobacter jejuni from New South Wales, Australia. J Glob Antimicrob Resist.

[62] Marotta F., Garofolo G., Di Marcantonio L., Di Serafino G., Neri D., Romantini R., Sacchini L., Alessiani A., Di Donato G., Nuvoloni R. (2019). Antimicrobial resistance genotypes and phenotypes of Campylobacter jejuni isolated in Italy from humans, birds from wild and urban habitats, and poultry. PLoS One.

[63] García-Fernández A., Dionisi A.M., Arena S., Iglesias-Torrens Y., Carattoli A., Luzzi I. (2018). Human campylobacteriosis in Italy: emergence of multi-drug resistance to ciprofloxacin, tetracycline, and erythromycin. Front Microbiol.

[64] Wieczorek K., Wołkowicz T., Osek J. (2018). Antimicrobial resistance and virulence-associated traits of Campylobacter jejuni isolated from poultry food chain and humans with diarrhea. Front Microbiol.

[65] Sierra-Arguello Y.M., Quedi Furian T., Perdoncini G., Moraes H.L., Salle C.T., Rodrigues L.B., Ruschel dos Santos L., Pereira Gomes M.J., Pinheiro do Nascimento V. (2018). Fluoroquinolone resistance in Campylobacter jejuni and Campylobacter coli from poultry and human samples assessed by PCR-Restriction fragment length polymorphism assay. PLoS One.

[66] Li Y., Zhang S., He M., Zhang Y., Fu Y., Liang H., Jing H., Li Y., Ma H., Zhang M. (2018). Prevalence and molecular characterization of campylobacter spp. isolated from patients with diarrhea in Shunyi, Beijing. Front Microbiol.

[67] Szczepanska B., Andrzejewska M., Spica D., Klawe J.J. (2017). Prevalence and antimicrobial resistance of Campylobacter jejuni and Campylobacter coli isolated from children and environmental sources in urban and suburban areas. BMC Microbiol.

[68] Cha W., Mosci R., Wengert S.L., Singh P., Newton D.W., Salimnia H., Lephart P., Khalife W., Mansfield L.S., Rudrik J.T. (2016). Antimicrobial susceptibility profiles of human Campylobacter jejuni isolates and association with phylogenetic lineages. Front Microbiol.

[69] Zhou J., Zhang M., Yang W., Fang Y., Wang G., Hou F. (2016). A seventeen-year observation of the antimicrobial susceptibility of clinical Campylobacter jejuni and the molecular mechanisms of erythromycin-resistant isolates in Beijing, China. Int J Infect Dis.

[70] Olkkola S., Nykäsenoja S., Raulo S., Llarena A.K., Kovanen S., Kivistö R., Myllyniemi A.L., Hänninen M.L. (2016). Antimicrobial resistance and multilocus sequence types of Finnish Campylobacter jejuni isolates from multiple sources. Zoonoses Public Health.

[71] Kim J., Lee M., Kim S., Jeon S.E., Cha I., Hong S., Chung G., Huh M.J., Kang Y.H., Yoo C.K. (2016). High‐level ciprofloxacin‐resistant Campylobacter jejuni isolates circulating in humans and animals in Incheon, Republic of Korea. Zoonoses Public Health.

[72] Abay S., Kayman T., Otlu B., Hizlisoy H., Aydin F., Ertas N. (2014). Genetic diversity and antibiotic resistance profiles of Campylobacter jejuni isolates from poultry and humans in Turkey. Int J Food Microbiol.

[73] Igimi S., Okada Y., Ishiwa A., Yamasaki M., Morisaki N., Kubo Y., Asakura H., Yamamoto S. (2008). Antimicrobial resistance of campylobacter: prevalence and trends in Japan. Food Addit Contam.

[74] Mukherjee P., Ramamurthy T., Bhattacharya M.K., Rajendran K., Mukhopadhyay A.K. (2013). Campylobacter jejuni in hospitalized patients with diarrhea, Kolkata, India. Emerg Infect Dis.

[75] Pollett S., Rocha C., Zerpa R., Patiño L., Valencia A., Camiña M., Guevara J., Lopez M., Chuquiray N., Salazar-Lindo E. (2012). Campylobacterantimicrobial resistance in Peru: a ten-year observational study. BMC Infect Dis.

[76] Liao C.-H., Chuang C.-Y., Huang Y.-T., Lee P.-I., Hsueh P.-R. (2012). Bacteremia caused by antimicrobial resistant campylobacter species at a medical center in Taiwan, 1998–2008. J Infect.

[77] Sonnevend Á., Kovács J., Pál T., Akawi N., Nagelkerke N., Schneider G. (2011). Lack of correlation between the 257C-to-T mutation in the gyrA gene and clinical severity of Campylobacter jejuni infection in a region of high incidence of ciprofloxacin resistance. Scand J Infect Dis.

[78] Said M.M., El-Mohamady H., Fawkia M., Rockabrand D.M., Ismail T.F., Monteville M.R., Ahmed S.F., Klena J.D., Salama M.S. (2010). Detection of gyrA mutation among clinical isolates of Campylobacter jejuni isolated in Egypt by MAMA-PCR. J Infect Dev Ctries.

[79] Kittl S., Kuhnert P., Hächler H., Korczak B. (2011). Comparison of genotypes and antibiotic resistance of Campylobacter jejuni isolated from humans and slaughtered chickens in Switzerland. J Appl Microbiol.

[80] Feodoroff F.B., Lauhio A.R., Sarna S.J., Hänninen M.L., Rautelin H.I. (2009). Severe diarrhoea caused by highly ciprofloxacin-susceptible campylobacter isolates. Clin Microbiol Infect.

[81] Rożynek E., Dzierżanowska-Fangrat K., Korsak D., Konieczny P., Wardak S., Szych J., Jarosz M., Dzierżanowska D. (2008). Comparison of antimicrobial resistance of Campylobacter jejuni and Campylobacter coli isolated from humans and chicken carcasses in Poland. J Food Protect.

[82] Bakeli G., Sato K., Kumita W., Saito R., Ono E., Chida T., Okamura N. (2008). Antimicrobial susceptibility and mechanism of quinolone resistance in Campylobacter jejuni strains isolated from diarrheal patients in a hospital in Tokyo. J Infect Chemother.

[83] Feizabadi M.M., Dolatabadi S., Zali M.R. (2007). Isolation and drug-resistant patterns of campylobacter strains cultured from diarrheic children in Tehran. Jpn J Infect Dis.

[84] Levesque S., Frost E., Michaud S. (2007). Comparison of antimicrobial resistance of Campylobacter jejuni isolated from humans, chickens, raw milk, and environmental water in Quebec. J Food Protect.

[85] Wardak S., Szych J., Zasada A.A., Gierczynski R. (2007). Antibiotic resistance of Campylobacter jejuni and Campylobacter coli clinical isolates from Poland. Antimicrob Agents Chemother.

[86] Unicomb L.E., Ferguson J., Stafford R.J., Ashbolt R., Kirk M.D., Becker N.G., Patel M.S., Gilbert G.L., Valcanis M., Mickan L. (2006). Low-level fluoroquinolone resistance among Campylobacter jejuni isolates in Australia. Clin Infect Dis.

[87] Sonnevend A., Rotimi V.O., Kolodziejek J., Usmani A., Nowotny N., Pál T. (2006). High level of ciprofloxacin resistance and its molecular background among Campylobacter jejuni strains isolated in the United Arab Emirates. J Med Microbiol.

[88] Guévremont E., Nadeau É., Sirois M., Quessy S. (2006). Antimicrobial susceptibilities of thermophilic campylobacter from humans, swine, and chicken broilers. Can J Vet Res.

[89] Cardinale E., Rose V., Perrier Gros‐Claude J.D., Tall F., Rivoal K., Mead G., Salvat G. (2006). Genetic characterization and antibiotic resistance of campylobacter spp. isolated from poultry and humans in Senegal. J Appl Microbiol.

[90] Norström M., Hofshagen M., Stavnes T., Schau J., Lassen J., Kruse H. (2006). Antimicrobial resistance in Campylobacter jejuni from humans and broilers in Norway. Epidemiol Infect.

[91] Lehtopolku M., Hakanen A.J., Siitonen A., Huovinen P., Kotilainen P. (2005). In vitro activities of 11 fluoroquinolones against 226 Campylobacter jejuni strains isolated from Finnish patients, with special reference to ciprofloxacin resistance. J Antimicrob Chemother.

[92] Boonmar S., Sangsuk L., Suthivarakom K., Padungtod P., Morita Y. (2005). Serotypes and antimicrobial resistance of Campylobacter jejuni isolated from humans and animals in Thailand. SE Asian J Trop Med Public Health.

[93] Tracz D.M., Keelan M., Ahmed-Bentley J., Gibreel A., Kowalewska-Grochowska K., Taylor D.E. (2005). pVir and bloody diarrhea in Campylobacter jejuni enteritis. Emerg Infect Dis.

[94] Gibreel A., Tracz D.M., Nonaka L., Ngo T.M., Connell S.R., Taylor D.E. (2004). Incidence of antibiotic resistance in Campylobacter jejuni isolated in Alberta, Canada, from 1999 to 2002, with special reference to tet (O)-mediated tetracycline resistance. Antimicrob Agents Chemother.

[95] Sharma H., Unicomb L., Forbes W., Djordjevic S., Valcanis M., Dalton C., Ferguson J. (2003). Antibiotic resistance in Campylobacter jejuni isolated from humans in the hunter region, New South Wales. Commun Dis Intell.

[96] Hakanen A.J., Lehtopolku M., Siitonen A., Huovinen P., Kotilainen P. (2003). Multidrug resistance in Campylobacter jejuni strains collected from Finnish patients during 1995–2000. J Antimicrob Chemother.

[97] Luber P., Wagner J., Hahn H., Bartelt E. (2003). Antimicrobial resistance in Campylobacter jejuni and Campylobacter coli strains isolated in 1991 and 2001-2002 from poultry and humans in Berlin, Germany. Antimicrob Agents Chemother.

[98] Gaudreau C., Gilbert H. (2003). Antimicrobial resistance of Campylobacter jejuni subsp. jejuni strains isolated from humans in 1998 to 2001 in Montreal, Canada. Antimicrob Agents Chemother.

[99] Bodhidatta L., Vithayasai N., Eimpokalarp B., Pitarangsi C., Serichantalergs O., Isenbarger D. (2002). Bacterial enteric pathogens in children with acute dysentery in Thailand: increasing importance of quinolone-resistant campylobacter. SE Asian J Trop Med Public Health.

[100] Sáenz Y., Zarazaga M., Lantero M., Gastañares M.J., Baquero F., Torres C. (2000). Antibiotic resistance in campylobacter strains isolated from animals, foods, and humans in Spain in 1997–1998. Antimicrob Agents Chemother.

[101] Aquino M., Filgueiras A., Ferreira M., Oliveira S., Bastos M., Tibana A. (2002). Antimicrobial resistance and plasmid profiles of Campylobacter jejuni and Campylobacter coli from human and animal sources. Lett Appl Microbiol.

[102] Thwaites R., Frost J. (1999). Drug resistance in Campylobacter jejuni, C coli, and C lari isolated from humans in north west England and Wales, 1997. J Clin Pathol.

[103] Li C.-C., Chiu C.-H., Wu J.-L., Huang Y.-C., Lin T.-Y. (1998). Antimicrobial susceptibilities of Campylobacter jejuni and coli by using E-test in Taiwan. Scand J Infect Dis.

[104] Luangtongkum T., Jeon B., Han J., Plummer P., Logue C.M., Zhang Q. (2009). Antibiotic resistance in campylobacter: emergence, transmission and persistence. Future Microbiol.

[105] Bukari Z., Emmanuel T., Woodward J., Ferguson R., Ezughara M., Darga N., Lopes B.S. (2025). The global challenge of campylobacter: antimicrobial resistance and emerging intervention strategies. Trop Med Infect Dis.

[106] Isada M., Reist M., MacKinnon M., Uhland F., Young K., Gibbens K., Parmley E., Carson C. (2022). Characterisation of burden of illness measures associated with human (fluoro) quinolone-resistant campylobacter spp. infections–a scoping review. Epidemiol Infect.

[107] Xia J., Pang J., Tang Y., Wu Z., Dai L., Singh K., Xu C., Ruddell B., Kreuder A., Xia L. (2019). High prevalence of fluoroquinolone-resistant campylobacter bacteria in sheep and increased campylobacter counts in the bile and gallbladders of sheep medicated with tetracycline in feed. Appl Environ Microbiol.

[108] Wieczorek K., Osek J. (2013). Antimicrobial resistance mechanisms among campylobacter. BioMed Res Int.

[109] Tang Y., Sahin O., Pavlovic N., LeJeune J., Carlson J., Wu Z., Dai L., Zhang Q. (2017). Rising fluoroquinolone resistance in campylobacter isolated from feedlot cattle in the United States. Sci Rep.

[110] van der Graaf-van Bloois L., Duim B., Looft T., Veldman K.T., Zomer A.L., Wagenaar J.A. (2023). Antimicrobial resistance in campylobacter fetus: emergence and genomic evolution. Microb Genom.

[111] Gazaigne L., Legrand P., Renaud B., Bourra B., Taillandier E., Brun-Buisson C., Lesprit P. (2008). Campylobacter fetus bloodstream infection: risk factors and clinical features. Eur J Clin Microbiol Infect Dis.

[112] Allos B. (2001). Campylobacter jejuni infections: update on emerging issues and trends. Clin Infect Dis.

[113] Lau C.L., Ramli R., Periyasamy P., Tan T.L., Neoh H-m, Yusof A.M., Zainal Abidin Z., Zulkifli Chia N., Mohd Saaid M.S., Abdul Aziz M. (2025;13:1-25). Performance of direct-from-blood-culture disk diffusion antibiotic susceptibility testing and its impact on antibiotic adjustment in bloodstream infections at a Malaysian tertiary center. Microbiol Spectr.

[114] Higgins J.P., Thompson S.G., Deeks J.J., Altman D.G. (2003). Measuring inconsistency in meta-analyses. BMJ.

[115] Egger M., Smith G.D., Schneider M., Minder C. (1997). Bias in meta-analysis detected by a simple, graphical test. BMJ.

[116] Yang Y., Feye K.M., Shi Z., Pavlidis H.O., Kogut M., Ashworth A.J., Ricke S.C. (2019). A historical review on antibiotic resistance of foodborne campylobacter. Front Microbiol.

[117] Mohan V., Strepis N., Mitsakakis K., Becker K., Chindelevitch L., Shivaperumal N., Swe-Han K.S., Hays J.P. (2025). Antimicrobial resistance in campylobacter spp. focussing on C. jejuni and C. coli–A narrative review. J Glob Antimicrob Resist.

[118] Park M., Kim J., Feinstein J., Lang K.S., Ryu S., Jeon B. (2022). Development of fluoroquinolone resistance through antibiotic tolerance in Campylobacter jejuni. Microbiol Spectr.

[119] World Health Organization (2022).

